# Prognosis Risk Model Based on Pyroptosis-Related lncRNAs for Bladder Cancer

**DOI:** 10.1155/2022/7931393

**Published:** 2022-02-02

**Authors:** Zechao Lu, Fucai Tang, Zhichen Li, Yongchang Lai, Zeguang Lu, Jiahao Zhang, Zhicheng Tang, Wanyan Cai, Zhaohui He

**Affiliations:** ^1^Department of Urology, The Eighth Affiliated Hospital, Sun Yat-sen University, Shennan Zhong Road #3025, Futian District, Shenzhen, Guangdong 518033, China; ^2^The Second Clinical College of Guangzhou Medical University, Guangzhou, Guangdong 511436, China; ^3^The Sixth Clinical College of Guangzhou Medical University, Guangzhou, Guangdong 511436, China; ^4^The Third Clinical College of Guangzhou Medical University, Guangzhou, Guangdong 511436, China; ^5^Department of Social and Behavioural Sciences, City University of Hong Kong, Hong Kong, China

## Abstract

**Objective:**

Bladder cancer (BC) is the most common malignancy in the urinary system and is prone to recurrence and metastasis. Pyroptosis is a kind of cell necrosis that is triggered by the gasdermin protein family. lncRNAs are noncoding RNAs that are more than 200 nucleotides long. Both pyroptosis and lncRNAs are associated with tumor development and progression. This study is aimed at exploring and establishing a prognostic signature of BC based on pyroptosis-related lncRNAs.

**Methods:**

In this study, The Cancer Genome Atlas (TCGA) database provided us with the RNA sequencing transcriptome data of bladder cancer patients, and we identified differentially expressed pyroptosis-related lncRNAs in bladder cancer. Then, the prognostic significance of these lncRNAs was assessed using univariate Cox regression analysis and LASSO regression analysis. Subsequently, 4 pyroptosis-related lncRNAs, namely, AL121652.1, AL161729.4, AC007128.1, and AC124312.3, were identified by multivariate Cox regression analysis, thus constructing the prognostic risk model. Then, we compared the levels of immune infiltration, differences in cell function, immune checkpoints, and m6A-related gene expression levels between the high- and low-risk groups.

**Result:**

Patients were divided into low-risk or high-risk groups based on the median risk score. Kaplan–Meier survival analysis indicated that the overall survival of bladder cancer patients in the low-risk group was substantially superior to that in the high-risk group (*p* < 0.001). The receiver operating characteristic (ROC) curve further confirmed the credibility of our model. Moreover, gene set enrichment analysis (GSEA) indicated that these were different signal pathways significantly enriched between the two groups. Immune infiltration, immune checkpoint, and N6-methyladenosine-related gene analysis also reflected that there were notable differences between the two groups.

**Conclusion:**

Therefore, this prognostic risk model is based on the level of pyroptotic lncRNAs, which is conducive to individualized assessment of the risk of patients and provides a reference for clinical treatment. This will also help provide insights into the prognosis and treatment of bladder cancer.

## 1. Introduction

Bladder cancer (BC) is the most common malignancy in the urinary system and the ninth most common cancer worldwide [1]. In the United States, more than 16,000 people die from bladder cancer each year [2]. At present, although transurethral resection of bladder tumors, radical cystectomy, pelvic lymphadenectomy, and other methods can be used for treatment, there are limitations such as neoplasm recurrence, neoplasm metastasis, and poor quality of life in postoperative patients. As a consequence, constructing an individualized prognostic risk model at the molecular level for bladder cancer patients is of tremendous significance in evaluating the risk level and carrying out treatment intervention promptly to improve the curative effect and prolong the survival time of BC patients.

Pyroptosis is a kind of cell necrosis that is triggered by the gasdermin protein family. Studies have shown that cytotoxic lymphocytes depend on pyroptosis to kill tumor cells, which are associated with the occurrence, development, and prognosis of bladder cancer [3]. In bladder cancer, the susceptibility of tumor cells to treatment is strongly linked with pyroptotic activity. That is, the more active pyroptosis in tumor cells, the better the patient prognosis. lncRNAs are noncoding RNAs that are more than 200 nucleotides long. Although without the ability to encode proteins, lncRNAs can not only regulate gene expression at epigenetic, transcription, and posttranscriptional levels but also function in many biological activities, including chromatin modification [4], transcriptional activation, and interference [5, 6]. In particular, lncRNAs can regulate the expression of pyroptosis-related genes and the activity of pyroptosis-related proteins [7]. As a result, lncRNAs play a crucial role in increasing or inhibiting tumor cell growth and spread. For example, knocking down lncRNA-XIST stopped non-small-cell lung cancer (NSCLC) from progressing by pyroptosis [8]. Moreover, exogenous overexpression of lncRNA GAS5 restrained ovarian cancer cell proliferation and colony formation by pyroptosis [9]. Nevertheless, the role pyroptosis-related lncRNAs play in bladder cancer has not been expounded. As a consequence, we tried to investigate the correlation between pyroptosis-related lncRNAs and bladder cancer.

Due to the rapid development of bioinformatics in recent years, sifting pyroptosis-related mRNA or lncRNA as prognostic signatures has become a reality, which is helpful to understand their role in the occurrence, development, and prognosis of carcinoma and provide new ideas for clinical prevention and treatment [10, 11]. In the current study, we downloaded and analysed a dataset of lncRNA expression in bladder cancer from The Cancer Genome Atlas (TCGA). Then, we filtered four pyroptosis-related lncRNAs that had considerable prognostic significance and built a prognostic model, which has great potential to independently predict the survival prognosis of bladder cancer patients.

## 2. Methods and Materials

### 2.1. Data Sources and Acquisition

TCGA was used to obtain the RNA sequence data and clinical information of BC. Expression and clinical information (including age, gender, stage, and prognosis) for RNA-seq (including mRNAs and lncRNAs) were obtained from TCGA database (https://tcga-data.nci.nih.gov/) on September 26, 2021. As the sequencing data from TCGA are publicly available, no further approval from the ethics committee is required. TCGA bladder cancer cohort included 411 tumor samples and 19 nontumor samples. Patients' samples were exempt from analysing if their follow-up information was not complete.

### 2.2. Identification of Pyroptosis-Related lncRNAs in Bladder Cancer

First, 33 pyroptosis-related encoding genes (mRNAs) were obtained from previous reviews [12–15]. Then, the expression level of 33 pyroptosis-related mRNAs was extracted from the expression matrix of TCGA by means of the limma package. Next, to identify pyroptosis-related differential lncRNAs, we used correlation tests (cor Filter = 0.4; *p* value Filter = 0.001) to screen lncRNAs related to pyroptosis-related mRNAs in bladder cancer. After that, we performed differential analysis on pyroptosis-related encoding genes and lncRNAs mentioned above, with the criteria of ∣log_2_ Fold Change (FC) | >0.5 and the False Discovery Rate (FDR) < 0.05.

### 2.3. Construction of a Prognostic Model for Bladder Cancer

After we obtained differentially pyroptotic lncRNAs, we conducted univariate Cox regression analysis, least absolute shrinkage and selection operator (LASSO) regression analysis, and multivariate Cox regression analysis to evaluate their prognostic value. Then, based on the above results, we selected the most representative candidates through LASSO analysis. Subsequently, these lncRNAs associated with survival were subjected to multivariate Cox regression analysis to construct the BC prognostic model. Moreover, on the basis of the median risk score, the patients were classified into one high-risk group and the other low-risk group.

(1) $$\begin{array}{c} \text{Risk scores}=\overset{n}{\underset{i=1}{\sum }}\left(\text{coef}\left(\text{lncRNA }i\right)*\text{Expr}\left(\text{lncRNA }i\right)\right). \end{array}$$

### 2.4. Evaluation of the BC Prognostic Model

First, we carried out Kaplan–Meier survival analysis to compare the survival probability and survival time between the high- and low-risk groups. Then, we applied ROC curves to assess the accuracy of the prognostic model in predicting bladder cancer patient survival. In addition, the t-distributed stochastic neighbor embedding (t-SNE) method was used to display the allocation of patients in different groups. Furthermore, Cox regression was used to determine whether patients' risk scores were an independent predictor for BC.

### 2.5. Nomogram Construction and Validation

A nomogram including clinical features such as age, sex, stage, and risk score obtained from prognostic signatures was constructed, which was able to analyse the survival probability at 1 year, 3 years, and 5 years. The range of total points was from 80 to 220.

### 2.6. Gene Set Enrichment Analysis (GSEA)

We conducted Kyoto Encyclopedia of Genes and Genomes (KEGG) pathway analysis to explain the differential signaling pathways of bladder cancer patients between the high-risk group and the low-risk group, thus deriving their biological functions.

### 2.7. Immune Infiltration, Immune Checkpoint, and N6-Methyladenosine-Related Gene Analysis

We downloaded the bladder cancer immune cell infiltration file from TIMER 2.0. Subsequently, limma and heat map packages were applied to generate a correlation heat map that visualized and compared the differences in immune cell infiltration between the high- and low-risk groups. Then, the “limma,” “GSVA,” “GSEABase,” “ggpubr,” and “reshape2” packages were utilized to perform ssGSEA, aimed at counting the immune cell infiltration scores and assessing the discrepancy in immunologic function between the high-risk group and the low-risk group. Furthermore, with the benefit of the “limma,” “reshape2,” “ggplot2,” and “ggpubr” packages, we detected the expression levels of immune checkpoint-related genes and N6-methyladenosine-related genes between the high-risk group and the low-risk group. Box plots were generated to visualize the differences.

### 2.8. Statistical Analysis

All of the statistical analyses in our study were performed using R version 4.1.1. We evaluated the correlation through Pearson correlation analysis and constructed survival curves through Kaplan–Meier analysis. Univariate and multivariate Cox regression analyses were utilized to examine the independence of our prognostic risk model in predicting the prognosis of bladder cancer patients. The ROC curve was used to assess our model's accuracy in predicting BC patient survival.

## 3. Result

### 3.1. Identification of Pyroptosis-Related lncRNAs and Construction of a Risk Score Model

**Figure 1:**
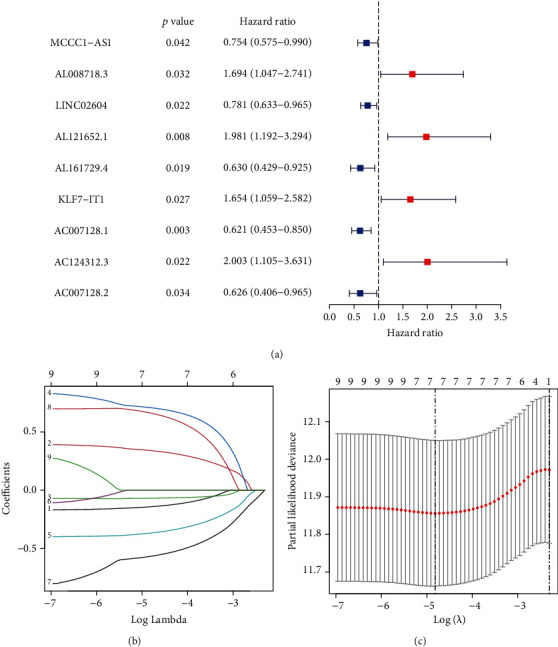
Identification of pyroptosis-related lncRNAs and construction of a risk score model. (a) The risk ratio forest plot indicated that 9 pyroptosis-related lncRNAs were associated with the overall survival of bladder cancer patients. (b) Adjustment of the parameters in LASSO regression analysis. (c) Diagram of the LASSO coefficient spectrum of prognostic pyroptosis-related lncRNAs.

Univariate Cox regression analysis showed that the expression of 9 pyroptosis-related differential lncRNAs, namely, MCCC1-AS1, AL008718.3, LINC02604, AL121652.1, AL161729.4, KLF7-IT1, AC007128.1, AC124312.3, and AC007128.2, was tightly associated with overall survival in bladder cancer patients. (Figure 1(a)). Then, we used LASSO regression analysis to reduce variables and select the most representative candidates for downstream analysis. The cross-validation verified that the optimum value in LASSO regression analysis was the best (Figures 1(b) and 1(c)). Subsequently, multivariate Cox regression analysis was applied to compute their respective coefficients, thus selecting the candidates, namely, AL121652.1, AL161729.4, AC007128.1, and AC124312.3, to construct the risk score model. The equation used for calculating the risk score of BC patients was as follows: Risk score = 0.925819034∗expr (AL121652.1)–0.549027307∗expr (AL161729.4)–0.673077592∗expr (AC007128.1) + 0.692429639∗expr (AC124312.3).

The former and latter of each item in the equation were regarded as the coefficient of pyroptosis-related lncRNAs through multivariate Cox regression analysis and the expression of pyroptosis-related lncRNAs, respectively.

### 3.2. Evaluation of the Prognostic Risk Score Model

**Figure 2:**
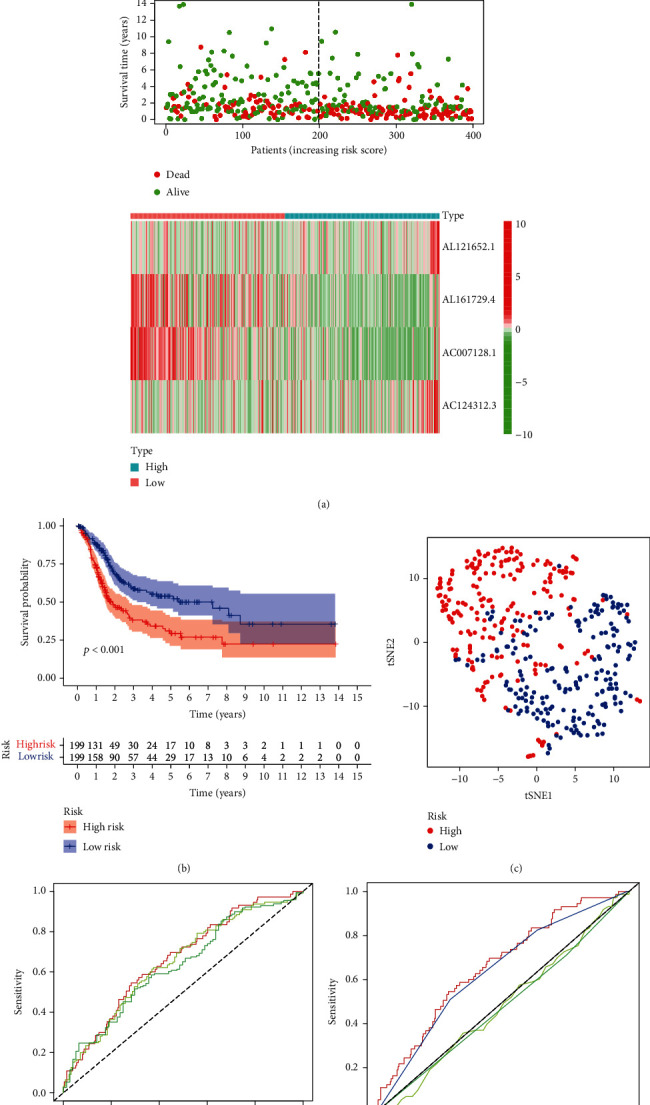
Evaluation of the pyroptosis-related lncRNA prognostic model for bladder cancer. (a) The risk curve consisted of risk scores in each sample, according to our prognostic risk model. The scatterplot consisted of survival status in each sample. The green dots represent alive, while the red dots represent dead. The heat map shows the expression levels of 4 pyroptosis-related lncRNAs in the low-risk group and high-risk group. (b) Kaplan–Meier survival analysis indicated that the survival time of bladder cancer patients in the high-risk group based on the pyroptosis-related lncRNA prognostic model was markedly shorter than that in the low-risk group. (c) t-SNE (t-distributed stochastic neighbor embedding) based on four pyroptosis-related lncRNAs showed two distinct distribution modes between the low- and high-risk groups. (d) The ROC curve indicated that the AUCs for 1-year, 2-year, and 3-year overall survival predicted were 0.663, 0.649, and 0.632, respectively. (e) The ROC curve showed that the pyroptosis-related lncRNA prognostic model was better than other predictive indicators, such as age, sex, and stage.

Based on the median risk score, the BC patients could be divided into two groups: high and low risk. The risk curve demonstrated a relationship between the risk score and risk level. Patients were ranked according to the pyroptosis-related lncRNA prognostic model. The higher the score, the greater the risk. A scatterplot revealed the relationship between the survival time and risk score of bladder cancer patients based on the pyroptosis-related lncRNA prognostic model. Both of them showed that the mortality of bladder cancer patients relied on their risk score (Figure 2(a)). In addition, Kaplan–Meier survival analysis indicated that the overall survival (OS) of bladder cancer patients in the low-risk group was substantially superior to that of patients in the high-risk group (*p* < 0.001) (Figure 2(b)). Moreover, t-SNE indicated two substantially distinct distribution modes between the high- and low-risk groups (Figure 2(c)). Finally, the AUC values for 1-year, 2-year, and 3-year overall survival were 0.663, 0.649, and 0.632, respectively (Figure 2(d)). The ROC curve analysis also proved that the four pyroptosis-related lncRNA signatures were a superior and reliable predictive model of prognostic value (AUC = 0.663) (Figure 2(e)).

### 3.3. Correlation of the Four Pyroptosis-Related lncRNA Signatures with Clinical Features

**Figure 3:**
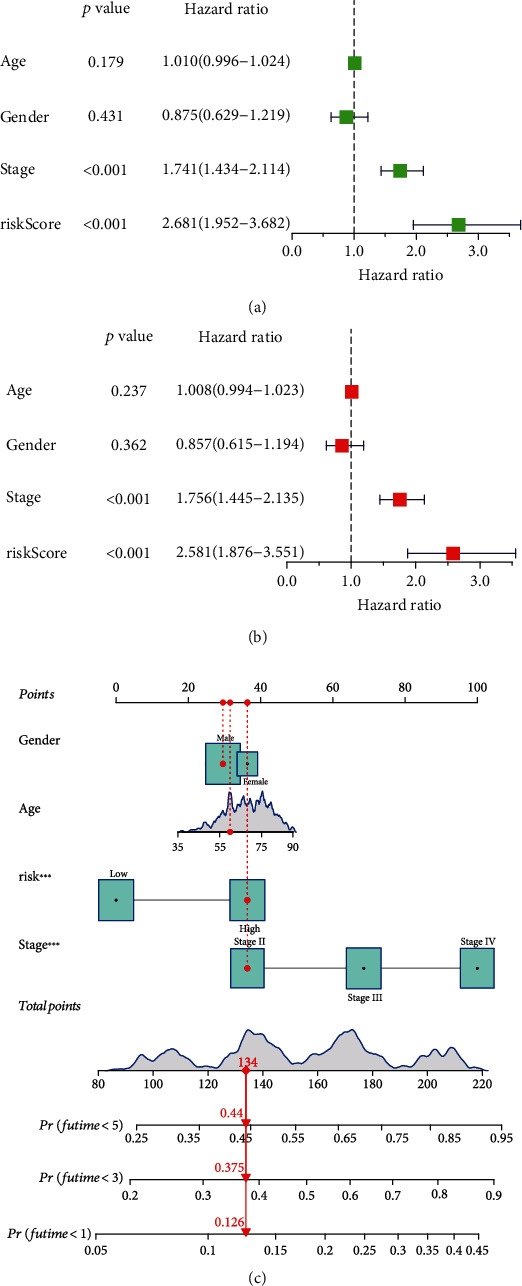
Correlation of the four pyroptosis-related lncRNA signatures with clinical features. (a) Univariate Cox regression analysis and (b) multivariate Cox regression analysis were applied to inspect whether the pyroptosis-related lncRNA prognostic model was independent of age, sex, and stage. (c) The nomogram included sex, age, risk score, and stage for predicting the 1-year, 3-year, and 5-year survival rates of bladder cancer patients.

**Figure 4:**
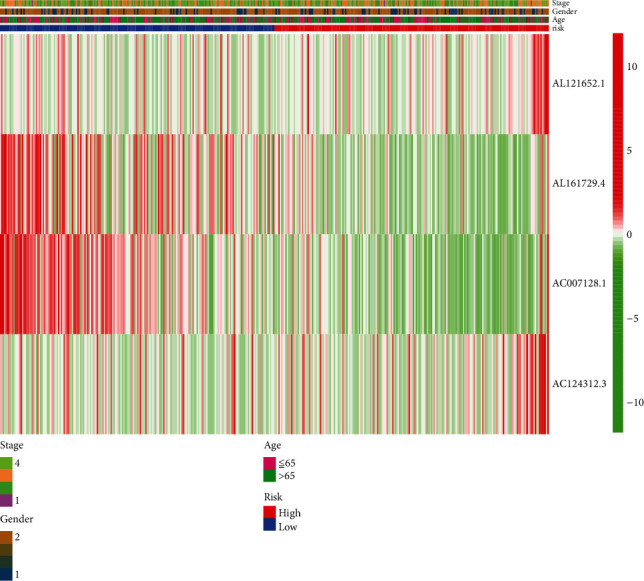
Heat map of the correlation between four pyroptosis-related lncRNAs and clinical features.

Univariate and multivariate Cox analyses of clinical features, including age, sex, stage, and risk scores, indicated that the four pyroptosis-related lncRNA signatures were independent prognostic factors (*p* < 0.001) (Figures 3(a) and 3(b)). We also devised a nomogram that included sex, age, risk score, and stage (Figure 3(c)). Furthermore, a heat map of the correlation between four pyroptosis-related lncRNAs, namely, AL121652.1, AL161729.4, AC007128.1, and AC124312.3, and clinical features, such as age, sex, and stage, was generated (Figure 4).

### 3.4. Gene Set Enrichment Analysis

**Figure 5:**
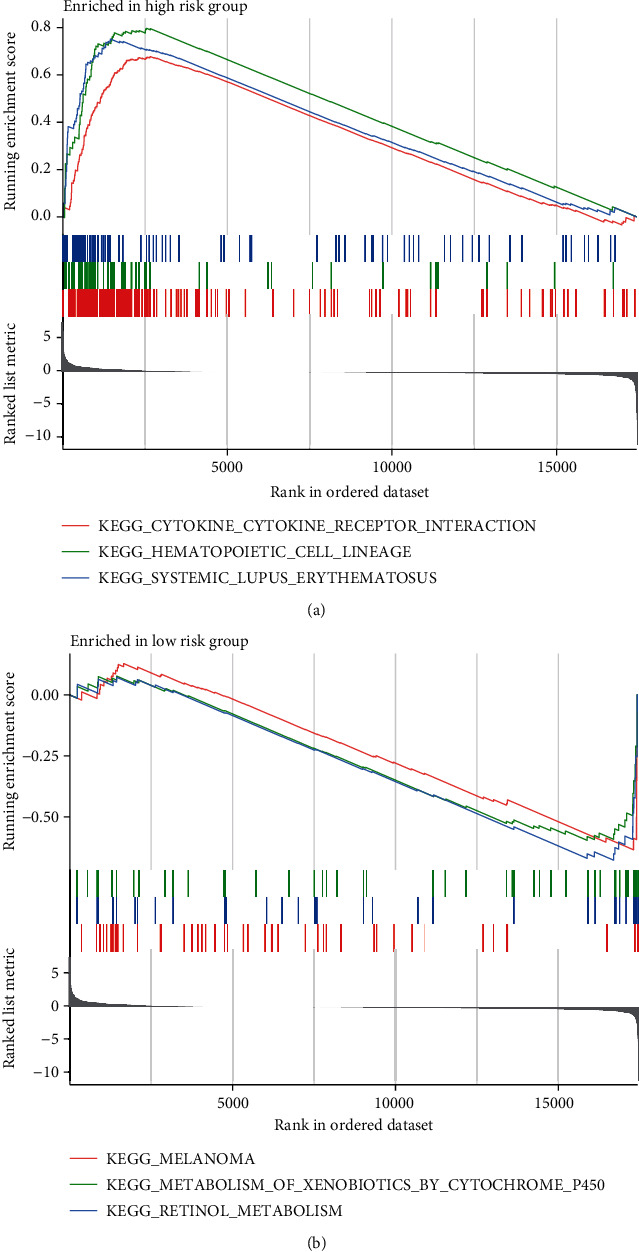
KEGG pathway analysis showed that four pyroptosis-related lncRNAs participated in the following physiological processes: cytokine cytokine-receptor interaction, hematopoietic cell lineage, systemic lupus erythematosus, melanoma, metabolism of xenobiotics by cytochrome, and retinol metabolism.

The KEGG pathway analysis showed that cytokine cytokine-receptor interaction, hematopoietic cell lineage, and systemic lupus erythematosus were enormously enriched in the high-risk group (Figure 5(a)), while melanoma, metabolism of xenobiotics by cytochrome, and retinol metabolism were substantially enriched in the low-risk group (Figure 5(b)). It is of tremendous significance for us to conduct further studies, based on the results mentioned above, on the mechanisms by which pyroptosis-related lncRNAs affect the occurrence, development, and prognosis of bladder cancer.

### 3.5. Immune Infiltration, Immune Checkpoint, and N6-Methyladenosine-Related Gene Analysis

**Figure 6:**
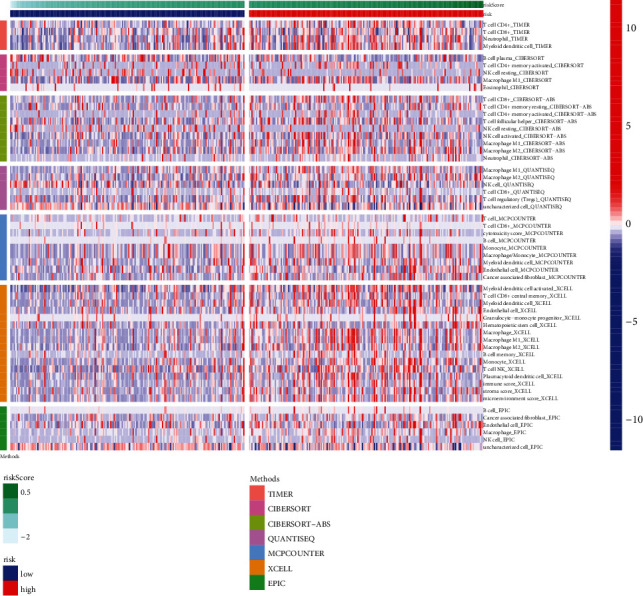
Heat map of immune cell infiltration analysis between the low- and high-risk groups of bladder cancer patients.

**Figure 7:**
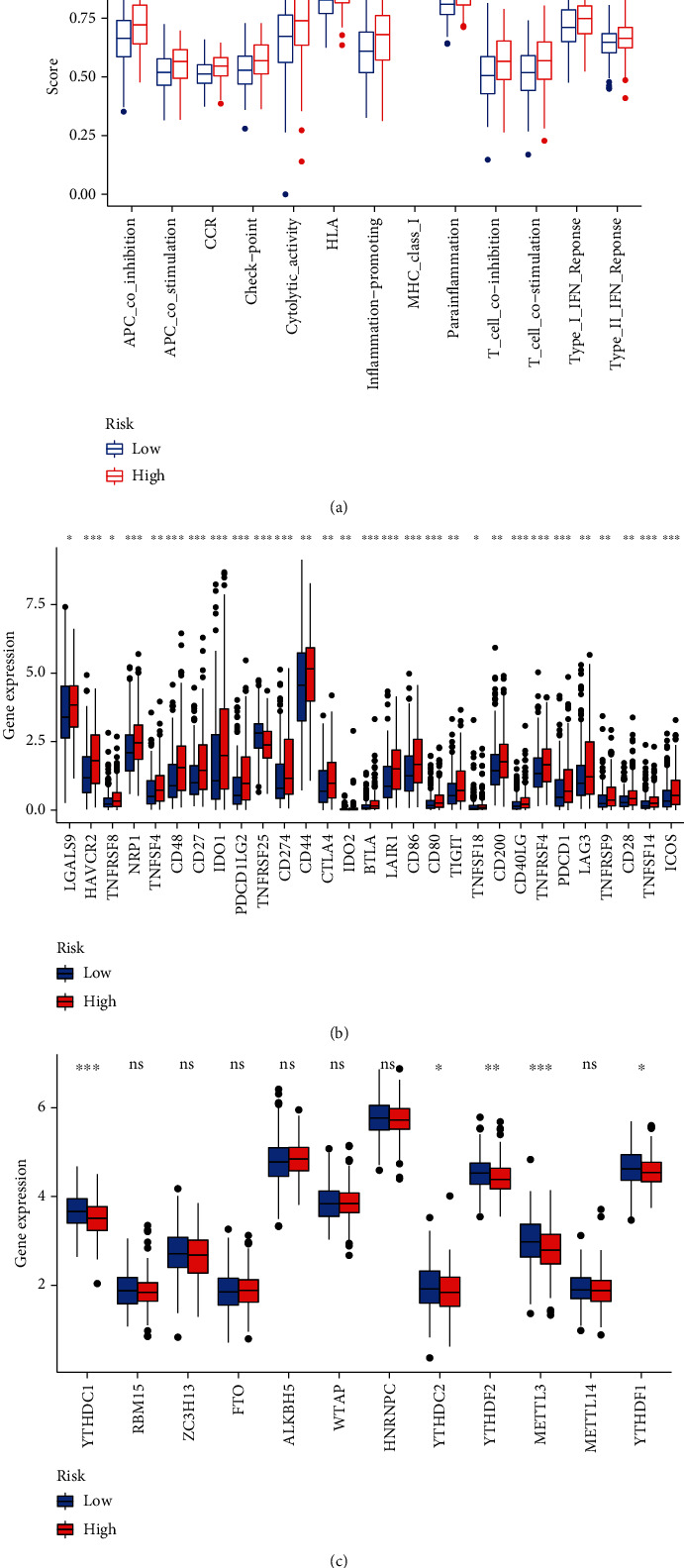
Discrepancy between the low-risk group and the high-risk group in terms of immunologic functions, immune checkpoint analysis, and N6-methyladenosine-related gene analysis. (a) The box plot of immunologic function analysis. APC: antigen-presenting cell; CCR: chemokine receptors; HLA: human leukocyte antigen; MHC class I: major histocompatibility complex class I; Type I IFN response: type I interferon response. (b) The box plot of immune checkpoint analysis. LGALS9: galectin 9; HAVCR2: hepatitis A virus cellular receptor 2; TNFRSF8: TNF receptor superfamily member 8; NRP1: neuropilin 1; TNFSF4: TNF superfamily member 4; CD48: CD48 molecule; CD27: CD27 molecule; IDO1: indoleamine 2,3-dioxygenase 1; PDCD1LG2: programmed cell death 1 ligand 2; TNFRSF25: TNF receptor superfamily member 25; CD274: CD274 molecule; CD44: CD44 molecule; CTLA4: cytotoxic T lymphocyte-associated protein 4; IDO2: indoleamine 2,3-dioxygenase 2; BTLA: B and T lymphocyte-associated; LAIR1: leukocyte-associated immunoglobulin like receptor 1; CD86: CD86 molecule; CD80: CD80 molecule; TIGIT: T cell immunoreceptor with Ig and ITIM domains; TNFSF18: TNF superfamily member 18; CD200: CD200 molecule; CD40LG: CD40 ligand; TNFRSF4: TNF receptor superfamily member 4; PDCD1: programmed cell death 1; LAG3: lymphocyte-activating 3; TNFRSF9: TNF receptor superfamily member 9; CD28: CD28 molecule; TNFSF14: TNF superfamily member 14; ICOS: inducible T cell costimulator. (c) Box plot of N6-methyladenosine-related gene analysis. YTHDC1: YTH domain-containing 1; RBM15: RNA-binding motif protein 15 ZC3H13: zinc finger CCCH-type containing 13; FTO: FTO alpha-ketoglutarate-dependent dioxygenase; ALKBH5: AlkB homolog 5, RNA demethylase; WTAP: WT1-associated protein; HNRNPC: heterogeneous nuclear ribonucleoprotein C; YTHDC2: YTH domain-containing 2; YTHDF2: YTH N6-methyladenosine RNA-binding protein 2; METTL3: methyltransferase 3, N6-adenosine-methyltransferase complex catalytic subunit; METTL14: methyltransferase 14, N6-adenosine-methyltransferase subunit YTHDF1: YTH N6-methyladenosine RNA-binding protein 1.

This study revealed significant variations in areas of immune response between the high-risk and low-risk groups, including immune cell infiltration, immunologic function, and immune checkpoint-associated genes. We also compared the difference in N6-methyladenosine-related genes between the two groups. The relative infiltration abundance of immune cells is shown in the heat map. Among them, CD8+ T cells, activated NK cells, macrophages, and myeloid dendritic cells infiltrated more in the high-risk group than in the low-risk group (Figure 6). In addition, the box plot of immunologic function analysis showed that immune reactions, specifically cytolytic activity and inflammation promotion, were significantly stronger in the high-risk group than in the low-risk group (Figure 7(a)). In addition, the box plot of immune checkpoint analysis revealed that, compared to low-risk group samples, the expression of representative immune checkpoint-related genes, including HAVCR2, IDO1, CD44, and LAIR1, was remarkably upregulated (Figure 7(b)). Finally, the box plot of N6-methyladenosine-related gene analysis illustrated that in the low-risk group, YTHDC1, YTHDF1, and METTL3 were downregulated in the high-risk group (Figure 7(c)).

## 4. Discussion

Bladder cancer is a common malignant tumor of the urinary system. Due to its high recurrence rate and easy metastasis, bladder cancer has become the ninth leading pathogeny of tumor-associated mortality around the world [16]. Annually, 3.0% of all newly diagnosed cancer cases and 2.1% of all cancer mortality can be attributed to bladder cancer [17]. Fortunately, thanks to the rapid development of high-throughput biological technology, predicting the prognosis of bladder cancer by their risk level assessment has become a reality. Based on the pyroptosis-related 4 lncRNAs listed above, we created a prognostic risk model that has been validated with considerable prognostic value in BC.

A number of recent studies have proposed some prognostic models for bladder cancer. Lu et al. constructed a 10 m6A-related ncRNA prognostic risk model for bladder cancer [18]. Xu et al. developed and validated a six-gene prognostic model for bladder cancer [19]. Yi et al. constructed an 8-gene model based on ferroptosis-related genes [20]. These models build risk models that can be used for bladder cancer based on different mechanisms. These models are beneficial for predicting the prognosis of bladder cancer patients and also help to deepen our understanding of the role of ferroptosis, m6A, and other mechanisms in bladder cancer. However, these models did not take into account the role of pyroptotic lncRNAs in bladder cancer, nor did they further consider the relationship between high-risk patients and immune infiltration, immune checkpoint genes, and so on. Based on the constructed pyroptosis lncRNA prognostic model, we also explored the differences in immune cell infiltration, immune function, immune checkpoint genes, and m6A-regulated genes among different risk groups. These results will help provide insights into the prognosis and treatment of bladder cancer.

Pyroptosis has been a hot topic in the field of cancer research. Considerable surveys show that pyroptosis plays a role in tumor cells and may act as a double-edged sword under different circumstances. For instance, the downregulation of GSDMD can significantly boost the proliferation and metastasis of gastric cancer cells [21], while the overexpression of GSDMB is related to reduced survival, easy invasion, and increased metastasis in breast cancer cells, predicting low sensitivity to targeted therapy [22]. As mentioned earlier, lncRNAs participate in the regulation of cell pyroptosis. Previous research has shown that pyroptosis-related lncRNAs can be used as novel biomarkers for NSCLC, ovarian cancer, and gastric cancer [8, 9, 23]. Nevertheless, there are few studies on pyroptosis-related lncRNAs in malignant tumors of the urinary system, especially for patients diagnosed with bladder cancer. Therefore, it is essential to carry out further investigation on pyroptosis-related lncRNAs in bladder cancer.

Considered new potential prognostic biomarkers, lncRNAs have gained widespread popularity in cancer. According to our study, among the four pyroptosis-related lncRNAs, AL161729.4 and AC007128.1 were protective factors, while AL121652.1 and AC124312.3 were risk factors. That is, the higher the expression of protective factors is, the better the prognosis of bladder cancer patients. The opposite is true for risk factors. To date, the four pyroptosis-related lncRNAs in our model have not yet been studied in bladder cancer. However, survival analysis performed by Ma et al. indicated that lncRNA AC124312.3 was notably correlated with the OS of triple-negative breast cancer patients and may be a promising therapeutic target [24]. Moreover, the upregulation of lncRNA AC007128.1 is associated with the poor prognosis of esophageal squamous cell carcinoma patients [25, 26]. To summarize, it is suggested that the four pyroptosis-related lncRNAs can be considered biomarkers to judge the prognosis of bladder cancer patients.

Recent studies have clarified the relationship between signaling pathways and pyroptosis. Functional enrichment analysis conducted by Luo et al. showed that cytokine cytokine-receptor interactions participate in the pathogenesis of bladder cancer [27]. Additionally, a meta-analysis showed that systemic lupus erythematosus was correlated with an increased risk for bladder cancers [28]. This result ties well with the KEGG pathway analysis in our study, where cytokine cytokine-receptor interactions and systemic lupus erythematosus were found to be markedly enriched in high-risk group patients. At the same time, these two pathways can regulate pyroptosis and are associated with the prognosis of BC. As a consequence, our study suggested that pyroptosis-related lncRNAs play a vital role in the occurrence, development, and prognosis of bladder cancer through the cytokine cytokine-receptor interaction and systemic lupus erythematosus signaling pathways.

Since pyroptosis-related lncRNAs are correlated with immune microenvironment [29], the risk level of bladder cancer patients may be potentially affected by immune infiltration. Zhang et al. found that high-risk group patients with hepatocellular carcinoma exhibited higher expression of immune checkpoint-related genes [30]. Moreover, Chen et al. found that m6A RNA methylation regulators, including METTL3, YTHDF2, and YTHDF1, were differentially expressed in bladder cancer tissues compared with normal tissues [31]. These results are aligned with the immune infiltration analysis in our study, where immune checkpoint- and N6-methyladenosine-related genes were found to be significantly upregulated in the high-risk group compared with the low-risk group. A further novel finding was that the more infiltration of immune cells, the worse prognosis of bladder cancer patients and so did immunologic functions. Consequently, immune infiltration plays a vital role in revealing the risk of bladder cancer. Further exploring the situation of immune infiltration will prove helpful in seeking the targets of bladder cancer immunotherapy.

However, we recognized that there are a few limitations and shortcomings in our study. First, the original data for constructing the pyroptosis-related lncRNA prognostic risk model were downloaded from TCGA. Moreover, biochemical experiments such as quantitative real-time PCR, immunohistochemistry, and flow cytometry must be designed to authenticate our model and further clarify the mechanism by which pyroptosis-related lncRNAs regulate the pathological process of bladder cancer.

## 5. Conclusion

To summarize, our study indicated that the prognostic risk model based on pyroptosis-related 4 lncRNAs can independently estimate the survival prognosis of BC. Furthermore, these 4 pyroptosis-related lncRNAs are potential prognostic biomarkers and may make outstanding contributions to seeking promising therapeutic targets for bladder cancer.

## Data Availability

The RNA-seq data and clinical follow-up data associated with the BC patient samples were downloaded from TCGA database (https://cancergenome.nih.gov/).
